# Breaking Rotational Symmetry in Supertwisted WS_2_ Spirals via Moiré Magnification of Intrinsic Heterostrain

**DOI:** 10.1021/acs.nanolett.2c03347

**Published:** 2022-11-08

**Authors:** Penghong Ci, Yuzhou Zhao, Muhua Sun, Yoonsoo Rho, Yabin Chen, Costas P. Grigoropoulos, Song Jin, Xiaoguang Li, Junqiao Wu

**Affiliations:** † Department of Materials Science and Engineering, University of California, Berkeley, California94720, United States; ‡ Materials Sciences Division, Lawrence Berkeley National Laboratory, Berkeley, California94720, United States; § Institute for Advanced Study, Shenzhen University, Shenzhen518060, China; ∥ Department of Chemistry, University of Wisconsin - Madison, Madison, Wisconsin53706, United States; ⊥ National Center for Electron Microscopy in Beijing, School of Materials Science and Engineering, Tsinghua University, Beijing100084, China; ¶ Department of Mechanical Engineering, University of California, Berkeley, California94720, United States; □ Physical & Life Sciences and NIF & Photon Sciences, Lawrence Livermore National Laboratory, Livermore, California94550, United States; ● School of Aerospace Engineering, Beijing Institute of Technology, Beijing, 100081, China

**Keywords:** twistronics, second harmonic generation, supertwisted spiral, symmetry breaking, moiré superlattice

## Abstract

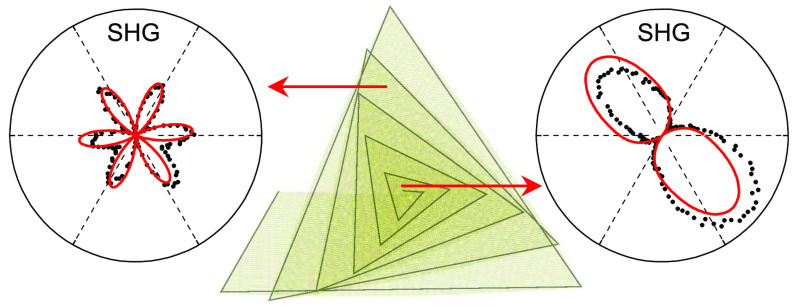

Twisted stacking of van der Waals materials with moiré superlattices offers a new way to tailor their physical properties via engineering of the crystal symmetry. Unlike well-studied twisted bilayers, little is known about the overall symmetry and symmetry-driven physical properties of continuously supertwisted multilayer structures. Here, using polarization-resolved second harmonic generation (SHG) microscopy, we report threefold (*C*_3_) rotational symmetry breaking in supertwisted WS_2_ spirals grown on non-Euclidean surfaces, contrasting the intact symmetry of individual monolayers. This symmetry breaking is attributed to a geometrical magnifying effect in which small relative strain between adjacent twisted layers (heterostrain), verified by Raman spectroscopy and multiphysics simulations, generates significant distortion in the moiré pattern. Density-functional theory calculations can explain the *C*_3_ symmetry breaking and unusual SHG response by the interlayer wave function coupling. These findings thus pave the way for further developments in the so-called “3D twistronics”.

Overlaying atomic layers of two-dimensional (2D) van der Waals (vdW) materials creates moiré superlattices, which have a microstructure controlled by the interlayer twist angle between adjacent layers. This strategy provides a fundamentally new paradigm for breaking and engineering crystal symmetries, thereby manipulating the internal quantum degrees of freedom and leading to numerous extraordinary physical phenomena.^1,2^ By controlling the spontaneous symmetry breaking in magic-angle moiré patterns, semimetallic bilayer graphene can be transformed into a succession of unconventional electronic phases, such as superconductors,^3^ quantum anomalous Hall Insulators,^4^ and Mott-like correlated insulators.^5^ In transition metal dichalcogenides (TMD) heterostructure superlattices, moiré excitons,^6−8^ Mott insulator states,^9^ and Wigner crystallization have been observed experimentally,^9^ along with modulation of the local atomic configuration with threefold (*C*_3_) rotational symmetry and energy extrema within the moiré supercell.^7^

Beyond well-studied twisted bilayers, interest in twisted multilayer systems has been growing, because the additional twist angles between each pair of neighboring layers offer a new degree of freedom to allow further manipulation of symmetry breaking and electron correlations.^10−12^ As a result of the interference between two bilayer moiré cells in the twisted multilayer systems, the formation of higher-order “moiré of moiré” leads to longer period superstructure which reveals more fascinating and robust physical properties.^13,14^ For example, recently, twisted trilayer and multilayer graphene have been reported to exhibit a broader range of magic angles and higher transition temperatures of superconductivity than twisted bilayer graphene.^15,16^ In TMD systems, compared to WS_2_/WSe_2_ heterostructure, graphene/WS_2_/WSe_2_ superlattices reportedly show deeper moiré potential and stronger interlayer coupling.^17^ Theoretical studies have revealed fascinating physical properties in continuously twisted multilayer systems, forming the basis for “3D twistronics”;^18,19^ for example, quantum geometry has been predicted to govern superconductivity and superfluidity in platforms including, but not limited to, twisted multilayer graphene.^10^ However, the “tear-and-stack” technique, the most prevalent way to fabricate twisted 2D structures, has limited capability and throughput for creating 3D twistronics.^20,21^ In light of this limitation, direct synthesis of continuously twisted TMD structures, termed “supertwisted spirals”, has been achieved via screw dislocation driven growth on non-Euclidean surfaces.^11^

The presence of screw dislocations and curved substrates disrupts the perfect crystalline periodicity, thereby inducing a native strain field in supertwisted spirals.^11,22^ A relative strain between adjacent twisted layers (heterostrain) can anisotropically affect superlattice wavelengths, known as the moiré magnification effect.^23−25^ This effect manifests as a dramatic distortion of the moiré pattern deviating from the hexagonal shape and hence leads to modifications of symmetry-related properties.^23,24,26,27^ Yet, the symmetry evolution in supertwisted spirals modulated by the combination of moiré superlattice and native strain remains unclear, impeding further understanding of their physical properties. Second harmonic generation (SHG) sensitive to electric dipoles in noncentrosymmetric systems is a powerful tool to probe symmetry-related physical properties that are otherwise challenging to study.^28,29^ Therefore, it is intriguing and essential to investigate whether supertwisted structures with intrinsic heterostrain possess abnormal symmetry breaking that can be probed by azimuthal polarization of SHG intensity. The answer is not straightforward, as tiny lattice deformation would not significantly change the symmetry of the system. For this reason, the traditional superposition theory of the second harmonic (SH) field would expect the SHG patterns of TMDs to be symmetric six petals with slight distortion when twist and strain are simultaneously tuned.^30−34^

Here, we report a *C*_3_ rotational symmetry breaking in supertwisted WS_2_ spirals revealed by polarization-resolved SHG, in contrast to the intrinsic *C*_3_ rotational symmetry of aligned WS_2_ layers. The relative strain between neighboring layers, demonstrated by the redshift of Raman peaks and multiphysics simulations, is magnified by the moiré superlattice and thus breaks the overall *C*_3_ rotational symmetry. Fundamentally, the overall symmetry breaking of electronic structure is caused by the strongly asymmetric charge density differences in the moiré supercell arising from a nontrivial interlayer coupling. We also developed a modified bond additivity model to simulate the effect of heterostrain and distorted moiré superlattice on the SHG patterns, which shows good agreement with the experimental SHG results for supertwisted spirals.

The synthesis and detailed structural characterization of the twisted WS_2_ spirals studied herein were described previously.^11^Figure 1a presents an atomic force microscopy (AFM) image of a “fastened” WS_2_ spiral with a right-handed twisted superstructure. This fastened spiral was grown on a protruded substrate, where the center of the screw dislocation sits above a protrusion denoted by the dashed line near the center of Figure 1a. Based on the non-Euclidean twist mechanism we previously reported,^11^ the crystal lattice twist is consistent with the geometric twist of the layers. Consequently, the shape of the spiral is bent following the protrusion’s curvature, which inevitably introduces nonuniform strain into the system. To explore the resultant potential symmetry modification of this spiral, we performed nonlinear SHG (2ω) characterization using a pulsed femtosecond laser with a fundamental wavelength (ω) of 800 nm (see details in Methods and Figure S1). Figure 1b shows the SHG microscopy image of the same area in Figure 1a by raster scanning the sample, indicating the breaking of inversion symmetry over the entire sample.

**Figure 1:**
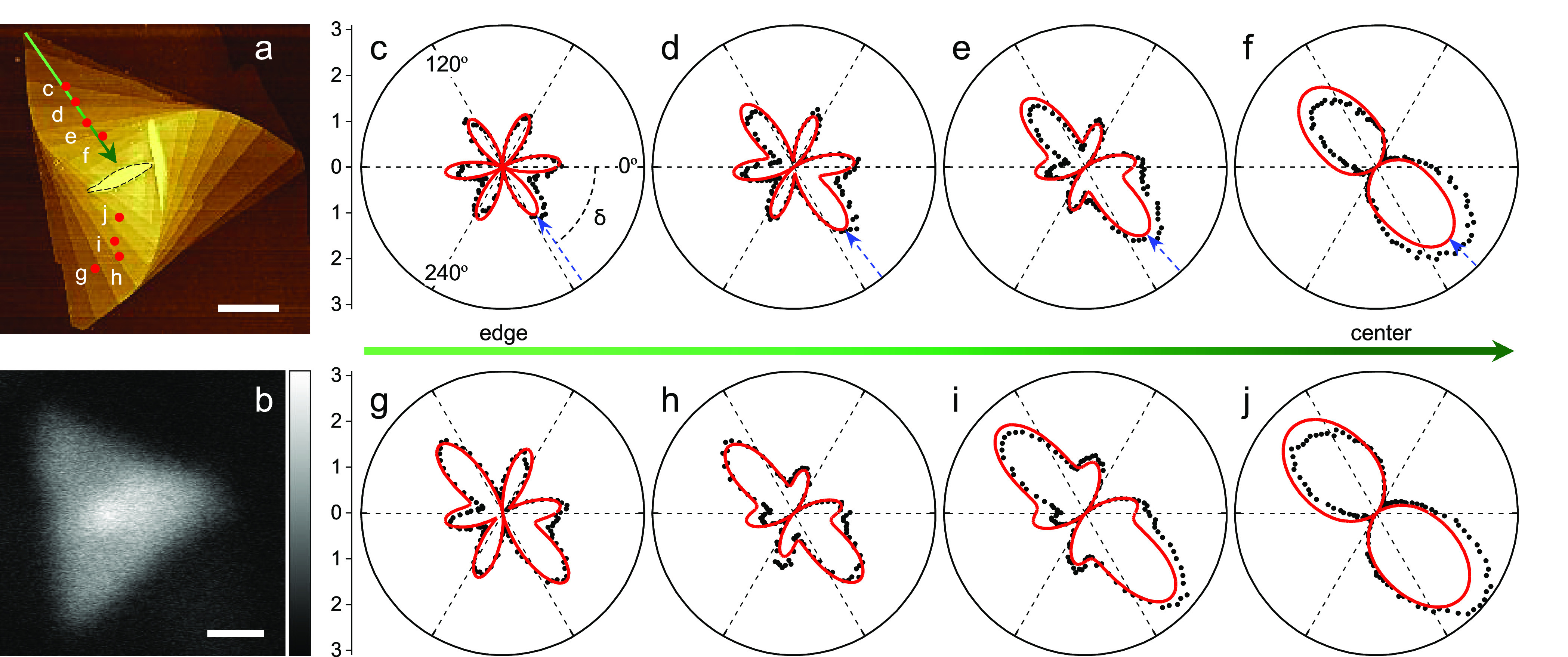
*C*_3_ rotational symmetry breaking near the center of a representative supertwisted WS_2_ spiral. (a) and (b) AFM and nonpolarized SHG intensity mapping images of a supertwisted WS_2_ spiral grown around a WO_*x*_ particle (marked by the black dashed line in panel (a) on SiO_2_/Si substrate (scale bar: 1 μm). (c)–(j) Polarization-resolved SHG intensity patterns at various positions labeled in panel (a), which gradually evolve from the six-petal into two-lobe symmetry along the green arrow. The incident laser and the SH electric field were linearly copolarized while rotating together relative to the sample plane. The azimuthal angle of 0° refers to incident laser polarization parallel to the horizontal direction in panel (a). Black symbols are experimental data; red curves are fitted via a modified bond additive model (Figure 5); blue arrows indicate the average “armchair” orientations at each measurement position.

After eliminating the impact of oblique incidence on SHG response (see Note 1 in SI), we further measured the azimuthal polarization-resolved SHG intensity patterns of this supertwisted WS_2_ spiral at different points of the supertwisted spiral (Figure 1c-j). Surprisingly, when approaching the spiral core, the SHG patterns transform from the normal six symmetric petals to an abnormal dumbbell shape. In stark contrast, aligned WS_2_ spirals grown on flat surfaces possess a 3R-like stacking geometry with an 0° interlayer twist, illustrated by the AFM image (Figure 2a,b) and previous studies,^35−37^ and exhibit undistorted six-petal polarization-resolved SHG intensity patterns everywhere (Figure 2d-f), independent of the measurement positions. This suggests that, unlike the aligned spiral, unexpected symmetry breaking occurs in the supertwisted spiral.

**Figure 2:**
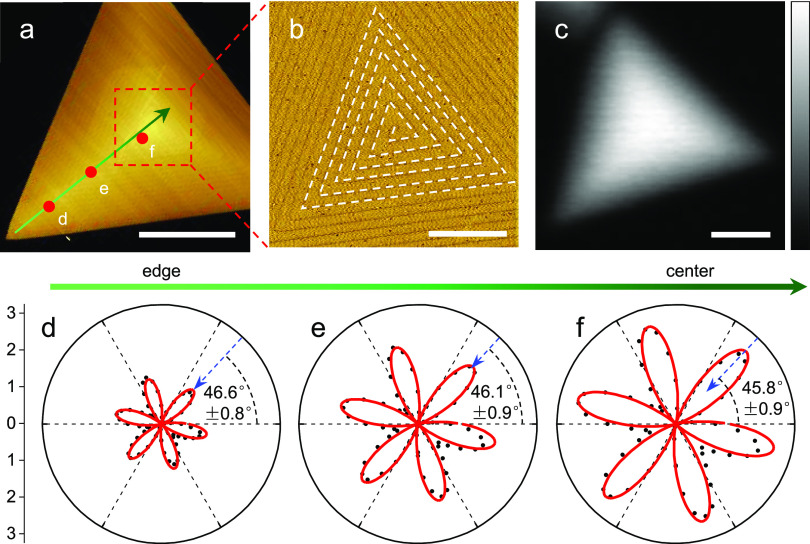
The persistence of *C*_3_ rotational symmetry in a representative aligned WS_2_ spiral. (a)–(c) AFM and nonpolarized SHG mapping images of an aligned WS_2_ spiral grown on a flat substrate. The zoomed-in AFM image in panel (b) shows the trace out of the dislocation spiral in the white dashed line. Scale bars: 2 μm, 0.5 μm, and 1.5 μm, respectively. (d)–(f) Polarization-resolved SHG intensity patterns at various positions labeled in panel (a), exhibiting a characteristic sixfold symmetry and thus confirming the *C*_3_ rotational symmetry in the entire sample. Black symbols are experimental data; red curves are fitted via SH field superposition theory; blue arrows indicate the armchair direction of the sample.

We start with analyzing the azimuthal polarization-resolved SHG intensity patterns by the superposition theory of the SH field,^33,38,39^ the most widely used model to fit the SHG response in twisted TMDs. Monolayer TMDs, belonging to the noncentrosymmetric *D*_3*h*_ point group, have a threefold rotation axis perpendicular to the surface.^30^ Thus, upon normal incidence, when the excitation polarizer is parallel to the SHG signal analyzer, the SHG intensity (*I*) pattern is known to exhibit sixfold rotational symmetry (Figure S1). This process is expressed as *I* ∝ |*E⃗*_2ω_|^2^ ∝ |cos 3ϕ|^2^, in which *E⃗*_2ω_ and ϕ are the SH electric field and the polarization angle of the laser, respectively.^30,40^ For stacked bilayers with a twist angle θ, the total SH field (*E⃗*_2ω_^*T*^) is the vector addition of dipole moments (*E⃗*_2ω_^1^ and *E⃗*_2ω_^2^) from two electrically decoupled layers: *E⃗*_2ω_^*T*^ = *E⃗*_2ω_^1^ + *E⃗*_2ω_^2^ ∝ cos 3ϕ + cos 3(ϕ + θ).^33,41^ The total SHG intensity of the twisted bilayers depends on the twist angle, but the SHG patterns preserve sixfold symmetry as reported in the literature.^33^ When expanding this theory to the twisted multilayers, the material absorption for the first to the *N*-th layer is taken into account to obtain the SH field from the *N-*th layer,^42^ and we found that the six-petal SHG pattern remains, as illustrated in simulations (Figure S2). Therefore, if only considering the degree of freedom of twist, the conventional SH field superposition theory cannot explain the two-lobe SHG patterns observed near the center of the supertwisted spirals. We need to note that, unlike the twisted structures fabricated by the “tear-and-stack” technique, the supertwisted spiral is natively deformed by the underlying protrusion during the growing process, thus introducing an additional degree of strain to contribute to symmetry-related physical properties.

To probe and estimate the strain magnitude, we measured the Raman spectra of supertwisted (Figure 3a,b) and aligned WS_2_ spirals (Figure 3c,d) at various points using a 488 nm laser excitation (see details in Methods). In the aligned spiral, both in-plane (E^1^_2g_) and out-of-plane (A_1g_) Raman vibrational modes monotonically rise from the edge to the center because of the increase in the thickness.^43^ In contrast, the supertwisted spiral exhibits a redshift of the E^1^_2g_ and A_1g_ peaks at positions near the center (Figure 3a,b), indicating the existence of nonuniform strain magnitude that compensates for the stiffened vibration caused by the increasing sample thickness.^44,45^ In the next step, using the reported A_1g_ Raman shift rates (∂ω(A_1g_)/∂ε) of 2H-stacked few-layer WS_2_ as a function of strain (∼0.74 cm^–1^/% strain),^45^ we estimate the tensile strain magnitude to be ∼0.4% around the position “f” in the supertwisted spiral. Note that in practice, the total strain must surpass 0.4% because the thickness-induced bond stiffening reduces strain-induced redshift of A_1g_ and the steric effect softens the atomic vibration for the randomly stacked TMDs, weakening the response of Raman mode to strain.^46^

**Figure 3:**
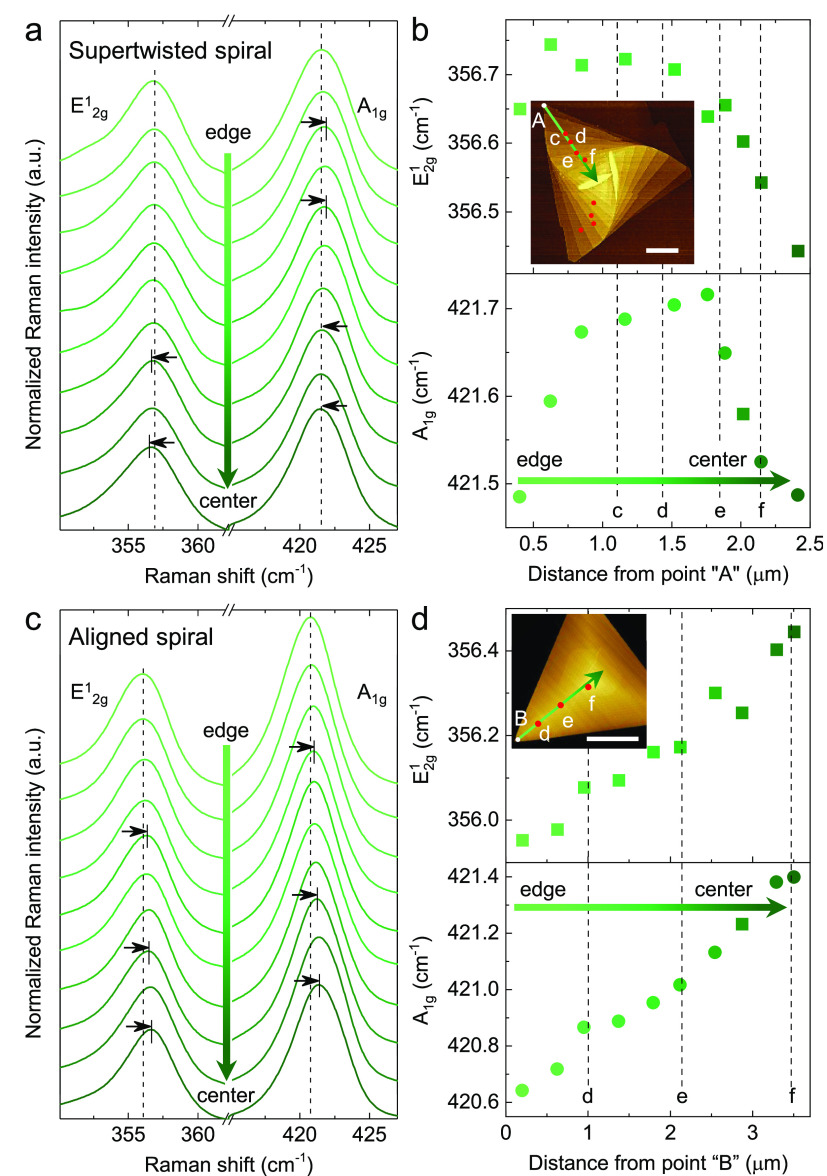
Probing strain magnitude in supertwisted WS_2_ spiral. (a) and (c) Raman spectra of a supertwisted and an aligned WS_2_ spiral along the green arrows in their respective AFM images (the insets in panels (b) and (d)). The dashed lines represent the peaks of the two phonon modes of the sample in the edge region. The black arrows show the redshift or blueshift of Raman peaks. (b) and (d) Individual E^1^_2g_ and A_1g_ mode frequencies of supertwisted and aligned WS_2_ spiral as a function of the distance from point “A” or “B” labeled in their respective AFM images (insets). The dashed lines indicate the positions where the SHG patterns were recorded in Figures 1 and 2. Scale bars for (b) and (d) are 1 and 2 μm, respectively. When approaching the center of the spiral, frequencies of both E^1^_2g_ and A_1g_ modes monotonically increase in the aligned spiral due to the increase in the number of WS_2_ layers. However, the frequencies drop near the center of the supertwisted spiral, indicating intrinsic and nonuniform strain in the supertwisted spiral.

We now turn to analyzing the role of strain in the SHG patterns of the supertwisted spiral. On the basis of the photoelastic effect, Mennel et al. demonstrated that the angular distribution of SHG intensity of monolayer TMDs under uniaxial strain is given by , where *A* and *B* are related to the magnitude of strain and the photoelastic coefficients, respectively, and γ is the principal strain orientation.^31,47^ Using a model based on a combination of Mennel’s equation and the SH field superposition theory, even when the tensile strain magnitude reaches as high as ∼3%, the SHG patterns would only become distorted and still retain the six-petal shape, as calculated in Figure S3. This can be attributed to two primary reasons: (1) Raman spectroscopy has limited capability in detecting the independent strain of each layer, whereas the heterostrain plays a critical role in moiré strain engineering and symmetry-related properties;^24,48−52^ (2) the theory of SH field superposition neglects the interlayer vdW interaction and models the SH response of individual layers as electrically decoupled layers, while interlayer hybridization of wave functions is known to modulate the overall symmetry and the resultant SHG patterns.^53,54^

The localized heterostrain in twisted TMD bilayers and their corresponding distorted moiré patterns have been observed using piezoresponse force microscopy.^49^ For vdW materials with thickness greater than five layers, however, it is challenging to experimentally quantify the strain of each layer. Therefore, we use COMSOL multiphysics to simulate the influence of protrusion on the formation of the supertwisted spiral by applying a force beneath the spiral. Interestingly, the deformation increases from the top layer to the bottom layer (Figure 4a,b), suggesting the presence of heterostrain in the supertwisted spiral. This can be understood by the mechanism of the “non-Euclidean” twist,^11,12,55^ in which the deformed upper layers tend to more easily relax via layer-to-layer sliding during the growing process of the supertwisted spirals because of the ultralow interfacial friction and weak interlayer forces in the vdW materials.^55,56^

**Figure 4:**
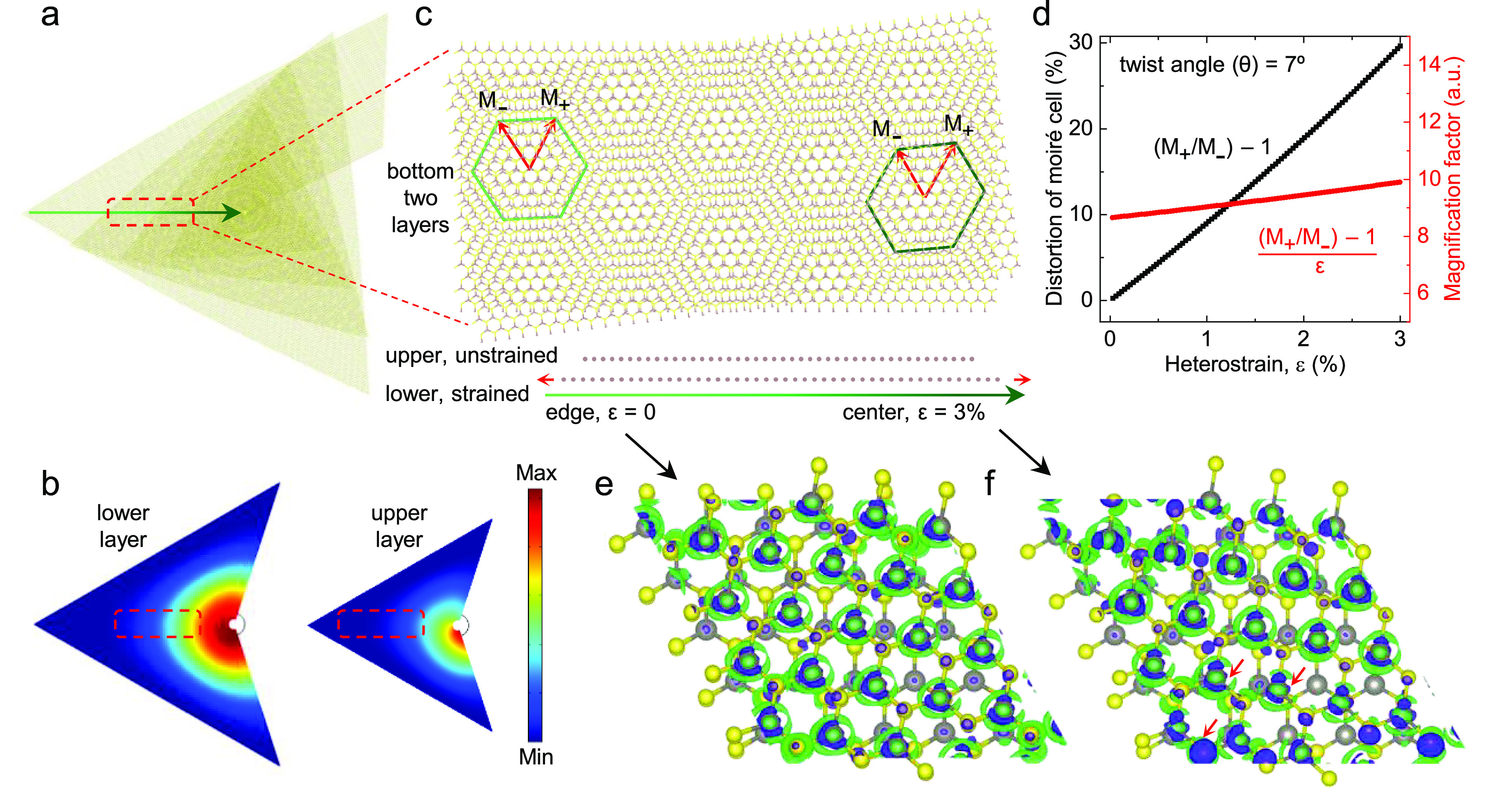
Symmetry transition induced by moiré magnification of intrinsic heterostrain in the supertwisted TMD spiral. (a) Schematic of a non-Euclidean supertwisted TMD spiral following the structure of the AFM image in Figure 1a. (b) Deformation of the bottom two layers qualitatively simulated by COMSOL (see details in Figure S4) increases from the edge of the spiral to its center. Compared to the lower layer, the tensile strain magnitude of the upper layer is so small that it is disregarded inside the red dashed rectangle, while the symmetry transition of the moiré pattern is investigated in (c). The upper layer is unstrained, but the lower layer undergoes enhanced strain (along the zigzag direction) from the edge to the center, leading to the broken *C*_3_ rotational symmetry of the moiré pattern (the dark green elongated hexagon), which is consistent with the two-lobe SHG patterns in Figure 1f,j. (d) Calculated length distortion (*M*_+_/*M*_–_ – 1) between two vectors of the moiré supercell and the magnification factor ((*M*_+_/*M*_–_ – 1)/ε) as a function of relative tensile strain (ε) between neighboring twisted layers. (e) and (f) DFT calculated charge density differences in the moiré supercell (twist angle of ∼7°) without strain and with tensile heterostrain of ∼3% (along zigzag direction), corresponding to the edge and the center of the spiral, respectively. Brown and yellow spheres represent W and S atoms, respectively. The blue and green areas represent electron accumulation and depletion due to the interlayer charge transfer. The red arrows show some localized regions with nonuniform distribution of charge density. The magnifying effect of the moiré pattern on heterostrain, combined with interlayer hybridization, strongly distorts the symmetry of charge density.

For twisted 2D lattices under heterostrain, an angular distortion in the lattice vectors anisotropically modulates the moiré pattern in each direction.^23^ As a result, the moiré pattern amplifies any small lattice deformation between neighboring layers, known as the magnifying glass effect,^23,24,26,27^ and the unit cells of the moiré superlattice can be distorted from hexagonal to oblique shape with a small heterostrain (Figures 4c and S5), modifying the lattice microstructure and consequently the electronic properties.^24^ The extension of such a theory from twisted bilayers to supertwisted multilayers is challenging, due to the higher-order “moiré of moiré” superlattice formation and the much larger incommensurate system size.^13,14,57^ Therefore, to simplify the treatment of a supertwisted structure, we investigate the moiré superlattice only in the bottom two layers of the supertwisted spiral, as illustrated by the dashed rectangle in Figure 4a,c. According to the numerically simulated deformation distribution, strain in the upper layer is negligibly small, but the lower layer experiences a higher, nonuniform strain, gradually increasing from the edge (ε = 0) to the center (assumed to be ∼3%) of the spiral. Owing to the low strain between the neighboring layers, the edge of the spiral retains *C*_3_ rotational symmetry (the light green hexagon in Figure 4c), in good agreement with the observed sixfold SHG patterns in Figure 1c,g. On the other hand, for the center of the supertwisted spiral, the heterostrain (ε) is magnified in the moiré superlattice by a factor of ∼(*M*_+_/*M*_–_ – 1)/ε. Here *M*_+_ and *M*_–_ are the two vectors of the moiré supercell (Figures 4c and S5) and are defined by , where *a* is the in-plane lattice constant of the monolayer vdW material, ϵ_±_ are related to the strain matrix, and φ_±_ are rotation angles of moiré vectors between twisted layers (see details in Figure S5).^23−25^ For the moiré pattern with an interlayer twist angle of 7° (the case of the supertwisted spiral sample in Figure 1a), a nearly 1 order of magnitude magnification of small heterostrains imposed by the moiré pattern (Figure 4d) would completely break the *C*_3_ rotational symmetry, as illustrated by the dark green distorted hexagon in Figure 4c (and Figure S5). This is in agreement with the observed two-lobe SHG patterns in Figure 1f,j.

We note that heterostrain-induced distortion of the moiré pattern in twisted systems is a necessary but not sufficient condition for obtaining a two-lobe SHG pattern.^53,54^ It is also important to consider interlayer coupling which arises from the hybridization of wave functions (particularly the sulfur orbitals) from neighboring layers to form bonding and antibonding states.^58,59^ Otherwise, the supertwisted spiral would inherit the band structure and SH fields from the individual layers, hence still generating a sixfold SHG pattern with some distortion, as predicted by the SH field superposition theory (Figure S3). To verify and explore the contribution of the interlayer coupling and charge transfer to electronic structure, we utilized density-functional theory (DFT) to calculate the charge density differences of an exemplary WS_2_ bilayer superlattice with an interlayer twist of 7° (Figure 4e,f). The unstrained moiré pattern presents a uniform electron accumulation and depletion (Figure 4e), consistent with the sixfold SHG patterns in the edge of the supertwisted spiral (Figure 1c). In stark contrast, for the bilayer superlattice with a heterostrain of ∼3% (Figure 4f), the distortion of the moiré pattern generates an uneven charge density redistribution (denoted by red arrows as examples) due to the interfacial charge transfer, which breaks the overall *C*_3_ rotational symmetry in the center of the supertwisted spiral and explains the observed two-lobe SHG patterns (Figure 1f,j). Indeed, following a similar reason, although SHG is forbidden for centrosymmetric monolayer graphene, exceptionally strong SHG is allowed in ABA-stacked trilayer graphene, originating from the overall inversion symmetry breaking combined with a nontrivial interlayer wave function interaction.^28^

Finally, to fit the SHG patterns of the supertwisted spiral (Figure 1c-j), we employ the bond additivity model (BAM) that is typically used to model the SHG response in monolayer TMDs distorted with one tilted bond.^60,61^ In the supertwisted spiral, the overall symmetry is modulated by the moiré magnification of heterostrain and results in a distorted moiré superlattice (Figure 4c,d), so a phenomenological approach can be employed by introducing an effective bond (Figure 5a) in the BAM to fit the *C*_3_ rotational symmetry breaking. The SHG intensity pattern is given by the modified BAM (see Note 2 in SI):
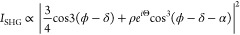
1where α is the angle of the additional bond away from the vertical W–S bond, δ is the angle between average “armchair” orientations of the supertwisted spiral and the horizontal direction (blue arrows in Figure 1), and ρ and Θ are the change in the amplitude and phase of the dipole moment of the effective bond, respectively. Equation 1 fits well with the measured SHG patterns in Figure 1c-j. Figure 5b presents that the values of the fitted angle δ rise linearly from the edge of the spiral to its center, showing good consistency with the values extracted from the SH field superposition theory (Figure S2), indicating a continuously twisted structure. The enhancement of ρ toward the center of the spiral suggests a complete breaking of the *C*_3_ symmetry for the SHG response.

**Figure 5:**
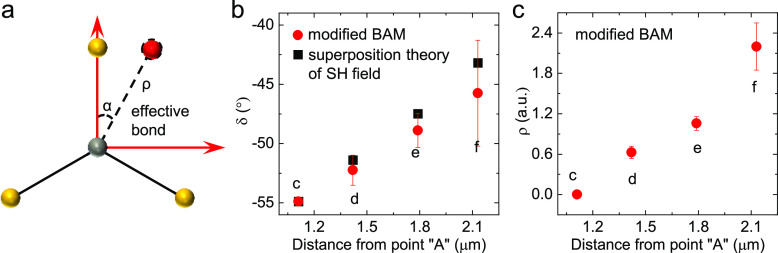
Modified bond additivity model (BAM) to fit SHG patterns of the supertwisted spiral. (a) Definition of coordinates for a triangular structural unit with an effective bond (red sphere) to derive the modified BAM (eq 1). Brown and yellow spheres represent W and S atoms, respectively. (b) and (c) Fitted parameters of δ (b) and ρ (c) as a function of distance for observed SHG patterns shown in Figure 1c-f.

In summary, we report a complete breaking of the original threefold rotational symmetry in supertwisted WS_2_ spirals arising from a nearly 1 order of magnitude magnification of small heterostrains between neighboring twisted layers by the moiré pattern. Combined with an interlayer hybridization and charge transfer effect, such symmetry breaking leads to a complete change of the polarization-resolved SHG pattern. Our results open new ways to engineering moiré superlattices of vdW materials by generating continuously twisted multilayer systems (3D twistronics), suggesting new avenues for exploring flat bands, moiré excitons, and strongly correlated insulating states.^17^ A wealth of intriguing symmetry-related physical effects are also potentially observed in the supertwisted spirals, such as enhanced intrinsic photovoltaic effect and in-plane anisotropy,^62,63^ as the *C*_3_ rotational symmetry breaking often results in anisotropic band structure and light–matter interactions.^1^ From a broad perspective, the *C*_3_ rotational symmetry breaking of the overall moiré pattern, together with the inversion symmetry breaking in individual TMD layers, may provide a general platform for studying various quantum geometrical phenomena, such as valley orbital magnetization,^64^ valley magnetoelectricity,^65^ and nonlinear Hall effects.^66,67^

## Supporting Information Available

More details for the sample synthesis and experimental setup; Figures S1–S5, Notes S1 and S2; SH field superposition theory; COMSOL multiphysics simulations; moiré magnification of heterostrain; modified bond additivity model (PDF)

## Abbreviations

SHG: second harmonic generation
*C*_3_: threefold
SH: second harmonic
2D: two-dimensional
vdW: van der Waals
TMD: transition metal dichalcogenides
AFM: atomic force microscopy
SH: second harmonic
DFT: density-functional theory
BAM: bond additivity model
