# Supplementary material for: Breaking Rotational Symmetry in Supertwisted WS2 Spirals via Moiré Magnification of Intrinsic Heterostrain
Source: Nano Lett. 2022 Nov 8;22(22):9027–35. doi: 10.1021/acs.nanolett.2c03347 (PMC9706673; doi:10.1021/acs.nanolett.2c03347)
Supplement: Supplementary file 1 — nl2c03347_si_001.pdf [file nl2c03347_si_001.pdf]

## Supporting Information

# Breaking rotational symmetry in supertwisted $WS_2$ spirals via moiré magnification of intrinsic heterostrain

Penghong Ci,[1,2,3,+] Yuzhou Zhao,[4,+] Muhua Sun,[5,+] Yoonsoo Rho,[6,7] Yabin Chen,[8] Costas P. Grigoropoulos,[6] Song Jin,[4] Xiaoguang Li,[3,*] Junqiao Wu[1,2,*]

[1] Department of Materials Science and Engineering, University of California, Berkeley, California 94720, United States

[2] Materials Sciences Division, Lawrence Berkeley National Laboratory, Berkeley, California 94720, United States

[3] Institute for Advanced Study, Shenzhen University, Shenzhen 518060, China

[4] Department of Chemistry, University of Wisconsin–Madison, Madison, WI 53706, United States

[5] National Center for Electron Microscopy in Beijing, School of Materials Science and Engineering, Tsinghua University, Beijing 100084, China

[6] Department of Mechanical Engineering, University of California, Berkeley, California 94720, United States

[7] Physical & Life Sciences and NIF & Photon Sciences, Lawrence Livermore National Laboratory, Livermore, California 94550, United States

[8] School of Aerospace Engineering, Beijing Institute of Technology, Beijing, China

[*] E-mail: wuj@berkeley.edu, xgli@szu.edu.cn

## Methods.

*Synthesis of $WS_2$ nanostructures.* The $WS_2$ spiral structures were synthesized following previous reports.[1, 2] $WO_3$ nanoparticles (Sigma Aldrich, nanopowder, <100 nm particle size) dispersed in ethanol were drop cast onto a 300 nm $SiO_2$/Si substrate and dried prior to the reaction. 100 mg $WS_2$ precursor powder (Alfa Aesar) in an alumina boat was placed in the first zone of a three-zone furnace. The substrates were placed between the second and the third zone with the polished side facing up. 1 g $CaSO_4{\cdot}2H_2O$ powder (Sigma Aldrich) was placed upstream from the heating zone as the water vapor source and heated with heating tapes. With 100 sccm argon flowing at 800 torr, the second zone was heated to 1200 °C at a rate of 20 °C/min, and simultaneously the third zone was heated to 700 °C at the same rate, while the $CaSO_4{\cdot}2H_2O$ was not heated. Once the furnace temperatures were reached, the $CaSO_4{\cdot}2H_2O$ was heated to 90 °C to release the water vapor. After all the temperatures were stabilized, the $WS_2$ boat was pushed into the second zone by a magnet coupled positioner and a quartz rod to initiate the reaction. After 15 min reaction, the furnace was opened and cooled down rapidly.

*Polarization resolved second harmonic generation (SHG) intensity patterns and imaging.* SHG measurement was performed using a Ti:Sapphire femtosecond laser with ~800 nm of wavelength, ~100fs of pulse width, and 80 MHz of repetition rate. The laser beam was mechanically chopped for signal demodulation to suppress the background noise. The polarization of the input laser beam was controlled by a half-wave plate and focused through a 50X long working distance objective lens. The generated SHG signal at ~400 nm was collected through the same objective lens and separated from the input fundamental laser beam by a harmonic separator. A polarizer filtered the polarization of the SHG signal, and color filters were used to block the fundamental laser beam. A photomultiplier tube (PMT) collected the SHG signal, which was demodulated at the modulation of the frequency of the input laser beam by a lock-in amplifier.

*Density-functional theory (DFT) calculations.* DFT calculations were performed using Vienna ab initio simulation package with projector-augmented wave pseudopotentials.[3, 4] The generalized gradient approximation of Perdew−Burke−Ernzerhof was used for the exchange-correlation functionals.[5] The cutoff energy for the plane-wave basis was set to 450 eV. The convergence criteria of energy and forces were set to $1{\times}10^{-4}$ eV and 0.05 eV $\text{Å}^{-1}$, respectively. The van der Waals interaction was induced by using the D3 correction scheme of Grimme.[6] For charge density difference calculations in twisted bilayer $WS_2$, we explored 4×4 supercell as discussed in previous studies.[7, 8] The bottom pristine monolayer $WS_2$ with $D_{3h}$ symmetry has zigzag crystal orientation aligned with $x$-axis; the top layer with ~3% strain is twisted clockwise by ~7° with respect to the bottom layer. The 4×4 grid of unstrained $WS_2$ primitive cell was combined with the corresponding size strained $WS_2$ to build the supercell of homostructure and allow relaxation for DFT calculations. The $k$-point sampling was obtained from the Monkhorst−Pack scheme with a 4×4×1 mesh.

*Raman and AFM characterization.* Raman spectra were measured by a Raman spectrometer (Renishaw Inc.) with a 488 nm laser as the excitation source. Sample morphology was characterized by an AFM system (Vistascope, Molecular Vista) in the tapping mode.

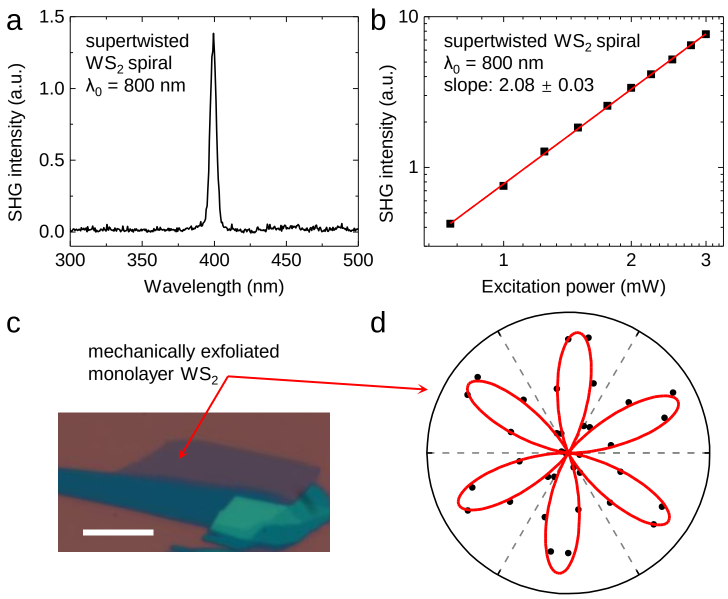


**Figure S1. SHG response of the supertwisted $WS_2$ spiral and monolayer $WS_2$.** (a) & (b) SHG signal intensity as a function of photon energy and excitation laser ($\lambda_0(\omega)$ = 800 nm) power in supertwisted $WS_2$ spiral. The SHG feature appears at the wavelength 400 nm (2ω), and its intensity grows quadratically with the incident power, verified by the slope of the fitted solid line to be 2.08 ±0.03. (c) & (d) Optical microscopy of mechanically exfoliated monolayer $WS_2$ (scale bar: 10 μm) and its polarization resolved SHG intensity pattern. Symbols are experimental data; curves are fitted via $I \propto |cos3\phi|^2$ (see details in the main text).

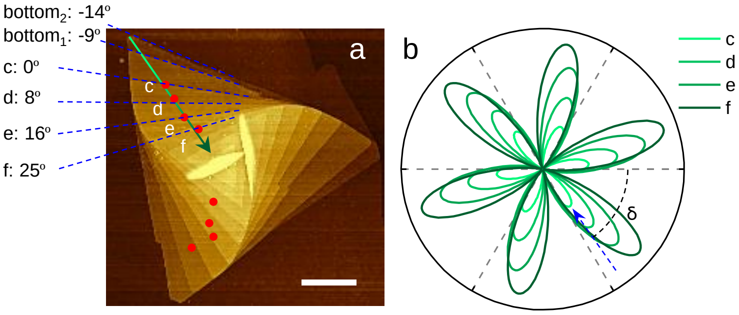


**Figure S2. Simulations of SHG patterns in the supertwisted $WS_2$ spiral based on SH field superposition theory considering the material absorption.** (a) AFM mapping image of the supertwisted spiral (scale bar: 1 μm), where the twist angles relative to the layer "c" are labeled. (b) Calculated SHG patterns in positions of "c" – "f" by SH field superposition theory show six-fold SHG patterns and thus cannot explain the experimental results in Figure 1. The blue arrow indicates the average "armchair" orientations ($\delta$) at the position of "c".

Based on the SH field superposition theory, for stacked bilayers with a twisting angle $\theta$, the total SH field ($\vec{E}^{T}_{2\omega}$) is the vector addition of dipole moments ($\vec{E}^{1}_{2\omega}$ and $\vec{E}^{2}_{2\omega}$) from two electrically decoupled layers: $\vec{E}^{T}_{2\omega} = \vec{E}^{1}_{2\omega} + \vec{E}^{2}_{2\omega} \propto cos3\phi + cos3(\phi + \theta)$.[9] 2H stacked bilayers ($\theta = 60^{o}$) yield a completely destructive interference of SH fields ($\vec{E}^{T}_{2\omega} = 0$), whereas $\theta = 0^{o}$, corresponding to the 3R-like stacking, yields a constructive superposition of SH fields. Hence the SHG intensity increases with the increasing number of 3R-like stacked layers,[10] explaining the enhancement of SHG intensity toward the center of the aligned spiral (Figure 2c).

When considering the material absorption, the SH field from the $N$th layer can be written as:[10]

$$\vec{E}^{N}_{2\omega} \propto \Gamma_N \cos\, 3(\phi - \delta_N) \quad \text{(S1)}$$

where $\Gamma_N = e^{i\Delta k(N-1)t} e^{-\alpha(N-1)t/2}$ defines the attenuation factor of the SH field from the $N$th layer due to the material absorption, $\phi$ is the azimuthal angle between the armchair direction and the incident laser polarization direction, $\alpha$ means absorption coefficient of $WS_2$ to be 0.086 $nm^{-1}$ at 400 nm,[11] $t$ is the thickness between the surface and the $N$th layer of the sample, $\delta_N$ is the twisting angle of the $N$th layer relative to the layer "c" denoted in the left of Figure S2a, $\Delta k = \frac{4\pi}{\lambda_0(\omega)}(-n(2\omega) - n(\omega))$ is the wave-vector difference between the fundamental ($\omega$) and the SH ($2\omega$) light, $\lambda_0$ is the wavelength of the fundamental laser to be 800 nm, $n(2\omega)$ and $n(\omega)$ mean the refractive index of $WS_2$ to be 3.78 at 400 nm and 4.10 at 800 nm,[12] respectively. According to the SH field superposition theory, the total SH field ($\vec{E}^{Total}_{2\omega}$) is the vector additions of dipole moments from each electrically decoupled individual layer ($\vec{E}^{N}_{2\omega}$):

$$\vec{E}^{Total}_{2\omega}(N, \delta) \propto \sum_{M=1}^{N} \Gamma_M \cos\, 3(\phi - \delta_M). \quad \text{(S2)}$$

Thus, the total SHG ($I^{Total}_{2\omega} \propto \left|\vec{E}^{Total}_{2\omega}\right|^2$) at different positions were calculated in Figure S2b. These patterns show full six-fold symmetry, indicating that the traditional SH field superposition theory cannot explain the experimental two-lobe SHG patterns in the supertwisted spiral (Figure 1). The average "armchair" orientations of $\delta$ fitted by SH field

superposition theory are consistent with those fitted by the modified bond additivity model in Figure 5b.

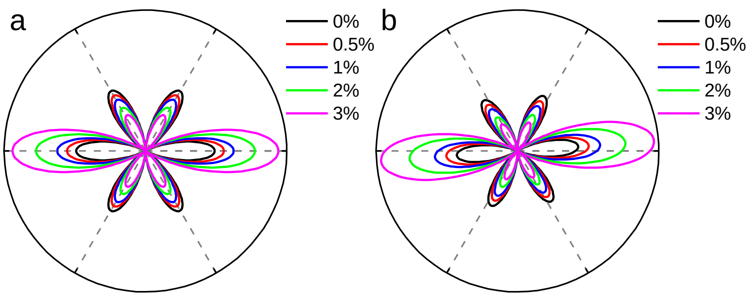


**Figure S3. Simulations of SHG patterns in $WS_2$ under strain.** (a) Calculated SHG patterns in monolayer $WS_2$ under various strains by Mennel's model. (b) Calculated SHG patterns in the supertwisted $WS_2$ spiral with the structure in Figure S2a as a function of strain, using a model based on a combination of Mennel's equation (eq S3), SH superposition theory, and the material absorption.

Mennel et al. demonstrated that SHG intensity patterns of monolayer TMDs under uniaxial strain are

$$I \propto \frac{1}{4}\left(Acos3\phi + Bcos(2\phi + \gamma)\right)^2 \text{ (S3)}$$

with $A = (1-\nu)(p_1 + p_2)(\varepsilon_{\mathrm{xx}} + \varepsilon_{\mathrm{yy}}) + 2\chi_0$ and $B = (1+\nu)(p_1 - p_2)(\varepsilon_{\mathrm{xx}} - \varepsilon_{\mathrm{yy}})$.[13] For $WS_2$, $p_1$ and $p_2$ are photoelastic parameters to be 0.75 and -0.97 nm/V/%, $\varepsilon_{\mathrm{xx}}$ and $\varepsilon_{\mathrm{yy}}$ are principal strains, $\chi_0$ is the nonlinear susceptibility parameter of the unstrained crystal lattice to be 7.5 nm/V, $\nu$ is the Poisson ratio to be 0.22, $\phi$ is the polarization angle, $\gamma$ denotes the principal strain orientation.[14] When setting $\gamma$ = 0º and $\varepsilon_{\mathrm{yy}}$ = 0, we calculated SHG patterns of monolayer $WS_2$ as a function of various $\varepsilon_{\mathrm{xx}}$ in Figure S3a. Considering the absorption-induced attenuation factor ($\Gamma_N$) of the SH field from the *N*th layer, we combined eqs S1-S3 to obtain the SHG intensity formula for the supertwisted spiral under uniform strain

$$I \propto \left|\sum_{M=1}^{N} \Gamma_M [Acos3(\phi - \delta_M) + Bcos(2(\phi - \delta_M) + \gamma)]\right|^2. \text{ (S4)}$$

Figure S3b shows SHG patterns of the supertwisted spiral with the structure in Figure S2a under uniform $\varepsilon_{\mathrm{xx}}$ strain ($\gamma$ = 0º, $\varepsilon_{\mathrm{yy}}$ = 0), which are inconsistent with experimental two-lobe SHG patterns in Figure 1.

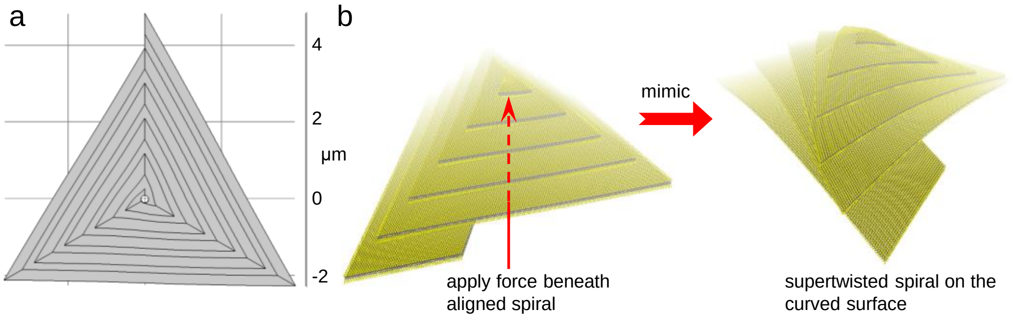


**Figure S4. COMSOL multiphysics simulations.** (a) COMSOL model of the spiral structure. (b) Sketch of a Euclidean aligned spiral and a non-Euclidean supertwisted spiral.

We determined the existence of heterostrain between adjacent layers in the supertwisted spiral structure using steady-state finite-element method (FEM) calculations (COMOSOL multiphysics with the Solid Mechanics module). Due to the challenge of building a supertwisted geometry above the curved surface, the regular spiral model was created in Figure S4a. An out-of-plane force was applied beneath the center of the aligned spiral to qualitatively mimic the influence of protrusion on the formation of the supertwisted spiral in Figure S4b, based on the mechanism of the “non-Euclidean” twist in our previous publication.[2] We note that as a result of the spiral shape in the three-dimensional space, its parallel cross section is not a complete triangle but a concave quadrilateral shape, as shown in Figure 4b. When applying the out-of-plane force beneath the spiral, the deformation increases from the upper layer to the lower layer (Figure 4b), suggesting the presence of heterostrain in the supertwisted spiral.

We note that the FEM simulations only qualitatively demonstrated the presence of distinct tensile strain among neighboring layers in the supertwisted structure, because real strain values in each layer are largely defined by the size of the protrusion and the relaxation process during the sample synthesis, which is challenging to be accurately simulated by COMSOL. However, real values of heterostrains in the supertwisted spirals do not affect the main conclusion of our work because a small heterostrain can be magnified about 10-fold, thus significantly distorting the moiré pattern and breaking the overall symmetry, as shown in Figure 4d.

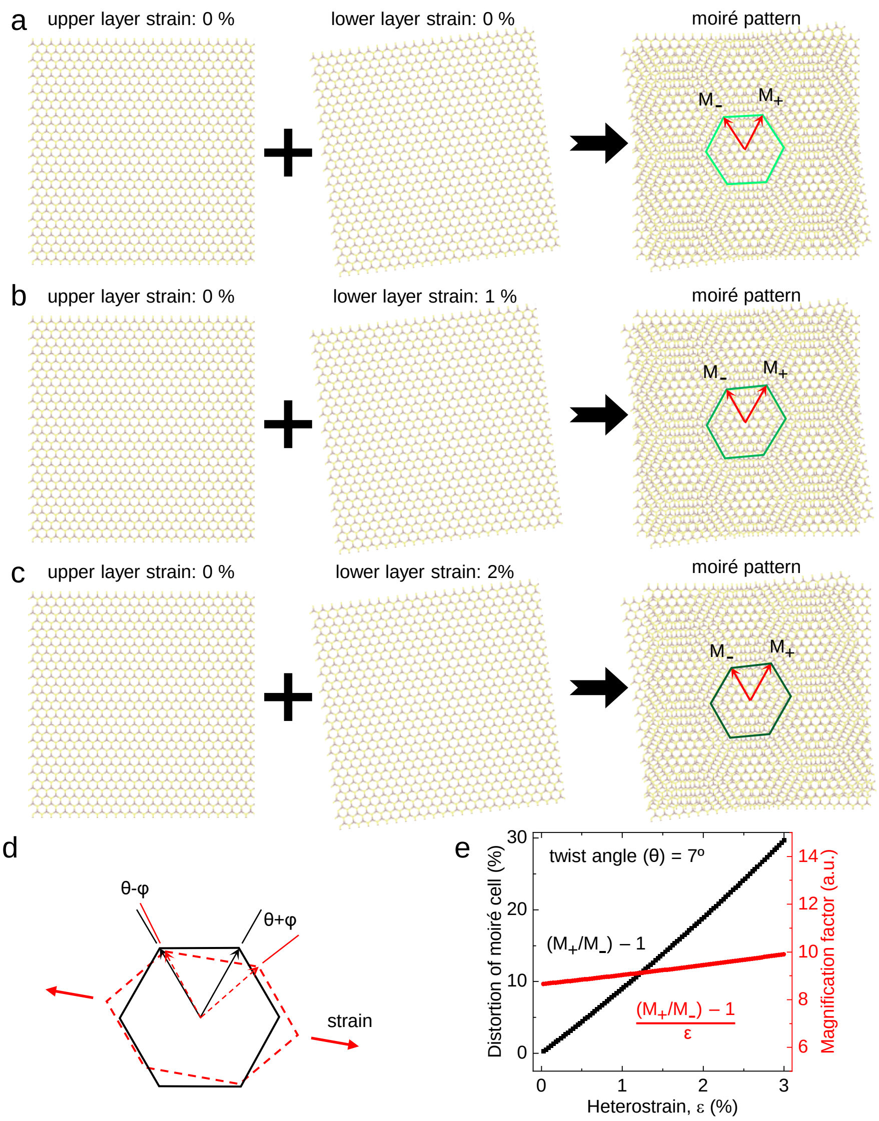


**Figure S5. Symmetry transition induced by moiré magnification of heterostrain in twisted TMD bilayers.** (a)-(c) The upper layer is unstrained, but the lower layer undergoes different strains of 0%, 1%, and 2%, hence forming moiré patterns with different shapes (twist angle of 7°). (d) Strained and rotated unit cell of the lower layer (dashed line) with respect to unstrained unit cell (solid line) in the upper layer.[15, 16] (e) Length distortion (M+/M- − 1) between two vectors of the moiré supercell and the magnification factor ((M+/M- − 1)/ε) as a function of relative strain (ε) between neighboring twisted layers.

The two vectors of the moiré supercell in Figures S5a-c are modulated by heterostrain to be

$$M_{\pm} = \frac{a(1+\epsilon_{\pm})}{2\sqrt{(1+\epsilon_{\pm})\sin^2[(\theta+\varphi_{\pm})/2]+\epsilon_{\pm}^2/4}}.$$ [15, 16] (S5)

For $WS_2$, the in-plane unit cell parameter is 0.315 nm; under a small strain approximation (ε << 1), we get $\epsilon_{\pm} \approx \frac{1}{4}(1-3\nu)\varepsilon$ and $\varphi_{\pm} \approx \pm\sqrt{3}\varepsilon\left|\frac{1+\nu}{4+\varepsilon(1-3\nu)}\right|$,[15, 16] where the Poisson ratio ($\nu$) is 0.22.[14] The twist angle ($\theta$) is tailored to $\theta + \varphi_{\pm}$ along two lattice vectors by heterostrain in Figure S5d.

When both layers have the same strain magnitude, the moiré pattern presents a regular hexagon shape, marked by the light green color in (a). The regular hexagonal moiré pattern is distorted into elongated hexagons for twisted TMD bilayers under heterostrain in (b)-(c).

**Note S1.** Eliminating the impact of oblique incidence on the SHG response.

The polarization resolved SHG patterns in Figure 1 were measured at positions away from the protrusion beneath the supertwisted spirals to ensure the normal incidence. Furthermore, due to the large ratio between the width (~200 nm) and the height (~0.7 nm) of the step, the stair angle of the spiral is too small to affect the SHG patterns. It has been reported that when the incident angle of the fundamental laser is $\eta$, the SHG intensity can be expressed as $I \propto |cos^2\eta \cdot cos3\phi|^2$.[17, 18] Thus, in our spiral structure, the $\eta$ value is so small (<< 1º) that the influence of oblique incidence on SHG is negligible.

**Note S2.** Modified bond additivity model to fit SHG patterns of the supertwisted spiral.

The polarization dependent SHG signal can be evaluated by the bond additivity mode (BAM).[19] Here, we consider a phenomenological mode by adding an effective bond to introduce the observed asymmetric two-fold signal in the SHG pattern.

The twist in the spiral structure will not affect the $D_{3h}$ symmetry of each $WS_2$ layer, so the 6-fold symmetry of the SHG pattern was initially expected to be preserved. However, our SHG signal exhibits a stronger angular preference closer to the center of the supertwisted spiral, where the heterostrain between neighboring twisted layers is enhanced. Based on the bond additivity model, the asymmetric phenomena can be explained by introducing an effective bond to describe the moiré magnification of heterostrain and the resultant elongated hexagon moiré patterns in Figures 5d and S5.

We assume the incident polarized light with the electric field as

$$\boldsymbol{E} = E_0 \hat{e} = E_0 (\sin\phi, \cos\phi, 0)^T$$

where $\phi$ is the angle between incident laser polarization and the y-axis of the lab coordinate, as shown in Figure 5a. For the $WS_2$ layer with $D_{3h}$ symmetry, the SHG comes from three effective linear bonds. Each of these bonds has the only nonzero hyperpolarizability tensor element $\beta_{yyy}^{(2)} = \beta_0$, where $y$ indicates the direction along this bond. Moreover, we consider an additional effective bond with an angle $\alpha$ relative to $y$-axis of the lab coordinate, and its $\beta_{yyy}^{(2)} = \beta_0 \rho e^{i\Theta}$. Now, in the lab coordinate, we have the total hyperpolarizability tensor

$$\beta_{lmn}^{(2)} = \beta_0 \rho e^{i\Theta} (\hat{l} \cdot \hat{\rho})(\hat{m} \cdot \hat{\rho})(\hat{n} \cdot \hat{\rho}) + \sum_i \sum_{lmn} \beta_{lmn}^{(2),i} (\hat{x} \cdot \hat{l})(\hat{x} \cdot \hat{m})(\hat{y} \cdot \hat{n})$$

where $\hat{\rho} = (\sin\alpha, \cos\alpha, 0)^T$ is the direction of the additional bond in the lab coordinate, $i$ stands for the different $WS_2$ bonds within the $D_{3h}$ symmetry bond, and $lmn$ indicate the bond coordinate. We note that the $z$ component of the effective bonds will not affect the SHG result.

The SHG intensity can be calculated as

$$P_i(2\omega) = \epsilon_0 \sum_{jk} \chi_{ijk}^{(2)} E_j E_k = \epsilon_0 \sum_{jk} 2 d_{ijk} E_j E_k$$

$$I_{SHG}(\phi) = \left| \epsilon_0 \sum_{ijk} E_i(2\omega) \chi_{ijk}^{(2)} E_j(\omega) E_k(\omega) \right|^2 = \left| \epsilon_0 \sum_{ijk} E_i(2\omega) 2 d_{ijk} E_j(\omega) E_k(\omega) \right|^2$$

$$d = \begin{bmatrix} \chi_{xxx} & \chi_{xyy} & \chi_{xzz} & \chi_{xyz} & \chi_{xzx} & \chi_{xxy} \\ \chi_{yxx} & \chi_{yyy} & \chi_{yzz} & \chi_{yyz} & \chi_{yzx} & \chi_{yxy} \\ \chi_{zxx} & \chi_{zyy} & \chi_{zzz} & \chi_{zyz} & \chi_{zzx} & \chi_{zxy} \end{bmatrix} \begin{bmatrix} \sin^2\phi \\ \cos^2\phi \\ 0 \\ 0 \\ 0 \\ 2\sin\phi\cos\phi \end{bmatrix}$$

For the above incident field, we can write the nonlinear susceptibility tensor as

$$d = \begin{bmatrix} \chi_{xxx} & \chi_{xyy} & 0 & 0 & 0 & \chi_{xxy} \\ \chi_{yxx} & \chi_{yyy} & 0 & 0 & 0 & \chi_{yxy} \\ 0 & 0 & 0 & 0 & 0 & 0 \end{bmatrix}$$

The $WS_2$ bonds are

$$\rho_1 = (0,1,0)^T$$

$$\rho_2 = \left(\sqrt{3}/2\,, -1/2, 0\right)^T$$

$$\rho_3 = \left(-\sqrt{3}/2, -1/2, 0\right)^T$$

We can obtain

$$\beta_{xxy}^{(2)} = \sum_i \sum_{lmn} \beta_{lmn}^{(i)} (\hat{x} \cdot \hat{l})(\hat{x} \cdot \hat{m})(\hat{y} \cdot \hat{n}) + \rho(\hat{x} \cdot \hat{\rho})(\hat{x} \cdot \hat{\rho})(\hat{x} \cdot \hat{\rho})$$
$$= \left[\rho e^{i\Theta} \sin^2 \alpha \cos \alpha - \frac{3}{4}\right] \beta_0$$

Similarly, we have

$$\beta_{xxx}^{(2)} = \rho e^{i\Theta} \sin^3 \alpha \ \beta_0$$

$$\beta_{yxx}^{(2)} = \beta_{xxy}^{(2)} = \left[\rho e^{i\Theta} \sin^2 \alpha \cos \alpha - \frac{3}{4}\right] \beta_0$$

$$\beta_{xyy}^{(2)} = \beta_{yxy}^{(2)} = \rho(\hat{x} \cdot \hat{\rho})(\hat{y} \cdot \hat{\rho})(\hat{y} \cdot \hat{\rho}) = \rho e^{i\Theta} \sin \alpha \ \cos^2 \alpha \ \beta_0$$

$$\beta_{yyy}^{(2)} = \rho(\hat{y} \cdot \hat{\rho})(\hat{y} \cdot \hat{\rho})(\hat{y} \cdot \hat{\rho}) = \left[\rho e^{i\Theta} \cos^3 \alpha + \frac{3}{4}\right] \beta_0$$

Finally, we obtain

$$I_{SHG}(\theta) = N\beta_0^2 \left|\rho e^{i\Theta} \cos^3(\alpha - \phi) + \tfrac{3}{4} \cos 3\phi\right|^2. \text{(S6)}$$

where $N$ is the number of atoms participating in the SH response. Close to the center region, the easy deformation nature and the overall $C_3$ rotational symmetry breaking response to the second harmonic measurement.

Table I: Fitted parameters of SHG patterns for different positions in the supertwisted spiral (Figure 1a).

| | δ (º) | δ+α (º) | ρ (a.u.) | Θ (º) |
|---|---|---|---|---|
| c | -54.9 | -30 | 0 | 1 |
| d | -52.5 | -40.3 | 0.62 | 95.4 |
| e | -48.9 | -45.2 | 1.06 | 105.7 |
| f | -45.8 | -45.9 | 2.2 | 130.2 |

## REFERENCES


1. Zhao, Y.; Jin, S. Controllable water vapor assisted chemical vapor transport synthesis of WS2–MoS2 heterostructure. *ACS Materials Letters* **2020,** 2, (1), 42-48.
2. Zhao, Y.; Zhang, C.; Kohler, D. D.; Scheeler, J. M.; Wright, J. C.; Voyles, P. M.; Jin, S. Supertwisted spirals of layered materials enabled by growth on non-Euclidean surfaces. *Science* **2020,** 370, (6515), 442-445.
3. Kresse, G.; Furthmüller, J. Efficient iterative schemes for ab initio total-energy calculations using a plane-wave basis set. *Physical review B* **1996,** 54, (16), 11169.
4. Blöchl, P. E. Projector augmented-wave method. *Physical review B* **1994,** 50, (24), 17953.
5. Perdew, J. P.; Burke, K.; Ernzerhof, M. Generalized gradient approximation made simple. *Phys. Rev. Lett.* **1996,** 77, (18), 3865.
6. Grimme, S.; Antony, J.; Ehrlich, S.; Krieg, H. A consistent and accurate ab initio parametrization of density functional dispersion correction (DFT-D) for the 94 elements H-Pu. *The Journal of chemical physics* **2010,** 132, (15), 154104.
7. Zhu, Z.; Cazeaux, P.; Luskin, M.; Kaxiras, E. Modeling mechanical relaxation in incommensurate trilayer van der Waals heterostructures. *Physical Review B* **2020,** 101, (22), 224107.
8. Naik, M. H.; Jain, M. Ultraflatbands and shear solitons in moiré patterns of twisted bilayer transition metal dichalcogenides. *Phys. Rev. Lett.* **2018,** 121, (26), 266401.
9. Hsu, W.-T.; Zhao, Z.-A.; Li, L.-J.; Chen, C.-H.; Chiu, M.-H.; Chang, P.-S.; Chou, Y.-C.; Chang, W.-H. Second harmonic generation from artificially stacked transition metal dichalcogenide twisted bilayers. *ACS nano* **2014,** 8, (3), 2951-2958.
10. Zhao, M.; Ye, Z.; Suzuki, R.; Ye, Y.; Zhu, H.; Xiao, J.; Wang, Y.; Iwasa, Y.; Zhang, X. Atomically phase-matched second-harmonic generation in a 2D crystal. *Light: Science & Applications* **2016,** 5, (8), e16131-e16131.
11. Hsu, C.; Frisenda, R.; Schmidt, R.; Arora, A.; De Vasconcellos, S. M.; Bratschitsch, R.; van der Zant, H. S.; Castellanos-Gomez, A. Thickness-dependent refractive index of 1L, 2L, and 3L MoS2, MoSe2, WS2, and WSe2. *Advanced optical materials* **2019,** 7, (13), 1900239.
12. Jung, G.-H.; Yoo, S.; Park, Q.-H. Measuring the optical permittivity of two-dimensional materials without a priori knowledge of electronic transitions. *Nanophotonics* **2019,** 8, (2), 263-270.
13. Mennel, L.; Furchi, M. M.; Wachter, S.; Paur, M.; Polyushkin, D. K.; Mueller, T. Optical imaging of strain in two-dimensional crystals. *Nature communications* **2018,** 9, 516.
14. Mennel, L.; Paur, M.; Mueller, T. Second harmonic generation in strained transition metal dichalcogenide monolayers: MoS2, MoSe2, WS2, and WSe2. *APL Photonics* **2019,** 4, (3), 034404.
15. Miller, D. L.; Kubista, K. D.; Rutter, G. M.; Ruan, M.; de Heer, W. A.; First, P. N.; Stroscio, J. A. Structural analysis of multilayer graphene via atomic moiré interferometry. *Physical Review B* **2010,** 81, (12), 125427.
16. Qiao, J.-B.; Yin, L.-J.; He, L. Twisted graphene bilayer around the first magic angle engineered by heterostrain. *Physical Review B* **2018,** 98, (23), 235402.
17. He, C.; Wu, R.; Qi, M.; Huang, Y.; Zhou, Y.; Zhang, S.; Zhao, Q.; Xu, X. Dispersion Property and Evolution of Second Harmonic Generation Pattern in Type-I and Type-II van der Waals Heterostructures. *ACS Applied Materials & Interfaces* **2021,** 13, (23), 27334-27342.
18. He, C.; Zhu, L.; Huang, Y.; Du, W.; Qi, M.; Zhou, Y.; Zhao, Q.; Xu, X. Accurately Controlling Angle-Resolved Second Harmonic Generation by Stacking Orders from a MoS2 Homobilayer. *The Journal of Physical Chemistry C* **2022,** 126, (25), 10584–10592.
19. Lu, F.; Sun, Z.; Wu, S.; Liu, W.-T. Bond additivity model for anisotropic second-harmonic generation from two-dimensional honeycomb lattices. *Opt. Lett.* **2020,** 45, (2), 268-271.
